# Association of well-being-centered leadership with burnout and professional fulfillment among physicians: mediating effects of autonomy support and self-valuation

**DOI:** 10.1108/LHS-01-2025-0001

**Published:** 2025-06-09

**Authors:** Anthony C. Waddimba, Jamile Ashmore, Megan E. Douglas, Linda M. Thompson, Colleen Parro, J. Michael DiMaio, Tait D. Shanafelt

**Affiliations:** Department of Surgery, Division of Surgical Research, Baylor University Medical Center, Dallas, Texas, USA; Research Institute, Baylor Scott and White Health, Dallas, Texas, USA, and Department of Medical Education, Texas A&M University College of Medicine, Dallas, Texas, USA; Office of Professionalism and Well-being, Baylor Scott and White-The Heart Hospital, Plano, Texas, USA, and Department of Medical Education, Texas A&M University College of Medicine, Dallas, Texas, USA; Research Institute, Baylor Scott and White Health, Dallas, Texas, USA; Department of Psychology, University of North Texas, Denton, Texas, USA, and Research Institute, Baylor Scott and White Health, Dallas, Texas, USA; Research Institute, Baylor Scott and White Health, Dallas, Texas, USA, and Academic Research Team, Baylor Scott and White-The Heart Hospital, Plano, Texas, USA; Division of Cardiothoracic Surgery, Baylor Scott and White-The Heart Hospital, Plano, Texas, USA; Research Institute, Baylor Scott and White Health, Dallas, Texas, USA, and Department of Biomedical Engineering, Texas A&M University College of Medicine, College Station, Texas, USA; Department of Medicine, Stanford University School of Medicine, Palo Alto, California, USA

**Keywords:** Physician burnout, Professional fulfillment, Autonomy support, Self-valuation, Well-being-centered leadership, Mediated structural equation model

## Abstract

**Purpose:**

This study aims to investigate autonomy support and self-valuation as potential mechanisms by which supportive leadership improves physician well-being. Supportive leadership is one of the strongest predictors of physician well-being. However, mechanisms by which leadership behavior influences well-being remain unknown. The authors hypothesized that autonomy support and self-valuation mediate this relationship.

**Design/methodology/approach:**

This was a cross-sectional survey-based study of physicians in a tri-hospital cardiovascular health system in southwestern USA. An anonymized multidimensional questionnaire comprising standardized and pre-validated measures of leadership behavior, self-valuation, autonomy support, fulfillment and burnout was e-mailed to 815 eligible physicians in February 2024. Hypothesized multivariable pathways were investigated via structural equation modeling.

**Findings:**

In total, 122 participants answered the survey, 99 providing complete responses. Respondents were 75.76% male, 54.54% aged 41 to < 65 years, 44.44% white, 21.21% Asian and 52.52% in practice for ≥ 15 years. Reliabilities of ordinal scales were all ≥ 0.700, and univariate correlations were in expected directions. Fully, 24.24% of respondents were burned out, 63.64% professionally fulfilled, 70.71% had high autonomy support and 55.56% high self-valuation. Indirect effects of leadership support on fulfillment and burnout, mediated via autonomy support and self-valuation, were more significant than direct effects. Findings supported the study hypothesis that leadership support improves fulfillment and reduces burnout among physicians partly by fostering autonomy and self-valuation.

**Originality/value:**

Autonomy support and self-valuation within physician teams are highlighted as factors whose improvement well-being-centered leadership training programs specifically should target.

## Introduction

In recent years, the well-being of physicians has been consistently worse than that of professionals or workers in other fields (Shanafelt *et al.*, 2012, 2015b, 2019b, 2021a, 2022). Contemporary changes to the work climate within health-care delivery organizations have contributed to progressive diminution of the joy in clinical practice (Shanafelt *et al.*, 2016; Rao *et al.*, 2017). Current burnout prevalence among US physicians is estimated to be 45.2%, with only 36% reporting high professional fulfillment (Shanafelt *et al.*, 2025). Burnout threatens the physician workforce’s mental health (Shanafelt *et al.*, 2011), productivity (Dewa *et al.*, 2014), satisfaction (Shanafelt *et al.*, 2009) and retention (Hamidi *et al.*, 2018; Shanafelt *et al.*, 2023; Collins *et al.*, 2024), as well as patient-care continuity (Geva *et al.*, 2017), safety (Shanafelt *et al.*, 2010), quality (Tawfik *et al.*, 2019; Hodkinson *et al.*, 2022), cost (Han *et al.*, 2019; Sinsky *et al.*, 2022) and satisfactoriness (Haas *et al.*, 2000) hampering health system efficiency (Shanafelt *et al.*, 2017). These consequences underscore the imperative for real-world solutions to the physician burnout epidemic (Bodenheimer and Sinsky, 2014; Gergen Barnett, 2017; Shin *et al.*, 2023; West *et al.*, 2016; Panagioti *et al.*, 2017; Shanafelt and Noseworthy, 2017). Investigating potentially modifiable factors that promote professional fulfillment and mitigate burnout could help to unearth actionable solutions.

Support from an effective team leader is known to be a significant predictor of well-being among physicians (Demmy *et al.*, 2002; Shanafelt *et al.*, 2015a; Dyrbye *et al.*, 2020, 2021b, Mete *et al.*, 2022; Tawfik *et al.*, 2023; Dyrbye *et al.*, 2024; Ashmore *et al.*, 2024). The recently proposed construct of well-being-centered leadership focuses on specific leadership behaviors that enhance engagement and professional fulfillment (Shanafelt *et al.*, 2021b). Well-being-centered leadership support has been linked to physicians’ burnout (Shanafelt *et al.*, 2015a; Dyrbye *et al.*, 2020, 2021b, Mete *et al.*, 2022; Tawfik *et al.*, 2023; Dyrbye *et al.*, 2024; Ashmore *et al.*, 2024), work satisfaction (Shanafelt *et al.*, 2015a; Dyrbye *et al.*, 2020; Tawfik *et al.*, 2023; Ashmore *et al.*, 2024), professional fulfillment (Mete *et al.*, 2022; Ashmore *et al.*, 2024), teamwork climate (Tawfik *et al.*, 2023) and intention to stay with one’s organization (Mete *et al.*, 2022; Ashmore *et al.*, 2024; Tawfik *et al.*, 2023). However, specific mechanisms or pathways through which supportive leadership influences burnout and professional fulfillment essentially remain unknown. This leaves a gap in our ability to design effective and efficient well-being-centered leadership training programs, especially in health-care systems where a single exclusive direct-report supervisor for each physician is not the norm (Ashmore *et al.*, 2024). The scarcity of empirical data on mechanism(s) by which leadership behaviors affect well-being leaves organizations making best guesses or using inefficient “shotgun” approaches. Physicians process perceptions of their workplace via filters like self-valuation (Trockel *et al.*, 2019), autonomy support (Walter and Lopez, 2008; Exworthy *et al.*, 2019; Waddimba *et al.*, 2020), personal–organization values alignment (Shanafelt *et al.*, 2021c) and/or perceived gratitude (Amano *et al.*, 2024). Indeed, self-valuation (Trockel *et al.*, 2021) and autonomy support (Moreau and Mageau, 2012; Lases *et al.*, 2018; Dyrbye *et al.*, 2021a) have each been found significantly associated with physician well-being. Thus, supportive leadership behavior plausibly can influence well-being outcomes directly and/or indirectly via mediating factors such as self-valuation, autonomy support, gratitude, feeling valued and others.

This study investigated whether an intrinsic factor, self-valuation, and an extrinsic factor, autonomy support, partially mediate the influence of well-being leadership support on burnout and professional fulfillment among physicians serving in a specialized cardiovascular hospital system. Self-valuation was defined as *deferment of self-care in favor of work demands plus harsh self-criticism in response to personal imperfections and errors* (Trockel *et al.*, 2019). Autonomy support was defined as *the free volition to use individual perspectives and exercise personal choices to perform clinical tasks without undue pressure* (Waddimba *et al.*, 2019) *while staying accountable* (Schumacher *et al.*, 2013). We hypothesized:

- that well-being-centered leadership support has a significant direct, negative association with burnout;
- a significant direct, positive association with fulfillment; and
- self-valuation and autonomy support mediate the effects of well-being-centered leadership on burnout and professional fulfillment.

The purpose of this research is to adduce evidence that could guide the development of effective strategies for coaching health-care leaders to focus their efforts on improving specific drivers of physician well-being.

## Methods and materials

### Study design

We investigated the hypotheses in a cross-sectional observational anonymized survey study. The study was approved by the Baylor Scott and White Research Institute institutional review board (IRB) under Protocol # 023–171. Prospective participants read a statement of the study aims, the strict anonymity of responses and the freedom to respond or not respond. Participants indicated their informed consent by choosing to answer the survey.

### Study participants

We sampled specialist and subspecialist physicians practicing in a three-hospital cardiovascular health-care system who responded to the 2024 annual *Physician Well-being Survey*. We excluded physicians still undergoing residency or fellowship training as their leadership and supervision structure significantly differs from that of specialists.

### Data collection

A hyperlink to the multidimensional online survey-questionnaire was distributed via e-mail to eligible participants on February1, 2024, then posters with QR codes were displayed in physician work areas. Participation remained open for two weeks, with weekly reminders sent via e-mail. Data were collected and managed using Research Electronic Data Capture (REDCap), a secure, Web-based electronic data capture tool hosted by our institution (Harris *et al.*, 2009, 2019).

#### Standardized and validated survey measures.

*Well-being-centered leadership support* was assessed using the nine-item Mayo Leadership Impact Index (MLII) version adapted for application in contexts with matrixed, flexible and multi-source leadership structures (Ashmore *et al.*, 2024). Each item (e.g. “Ensures that I am treated with respect and dignity”) rates a leader on a five-point Likert spectrum ranging from 1 (“Strongly Disagree”) to 5 (“Strongly Agree”). The adapted MLII is scored by summing up constituent items so that higher total scores (minimum = 9; maximum = 45) indicate greater leadership support, and vice versa. Specific adaptations made to the original measure have been previously reported (Ashmore *et al.*, 2024), and the adapted stem question defined a leader as “the person or group considered most directly responsible for providing professional administrative guidance, feedback, and support.”

*Professional fulfillment* was assessed using the Professional Fulfillment Index’s (Trockel *et al.*, 2018) six-item professional fulfillment subscale (PFS). Each item asks respondents to rate feelings toward work in the preceding two weeks (e.g. “I am contributing professionally in the ways I value most”) on a Likert spectrum ranging from 0 (“not at all true”) to 4 (“completely true”). The scale is scored by summing up then averaging constituent item scores (minimum score = 0; maximum score = 4). PFS scores ≥ 3.0 were deemed as indicating “high” professional fulfillment.

*Burnout* was assessed using the Professional Fulfillment Index’s (Trockel *et al.*, 2018) ten-item Overall Burnout Subscale (OBS). The OBS uses four *work exhaustion* items (e.g. “a sense of dread when I think about work I have to do”) and six *interpersonal disengagement* items (e.g. “less sensitive to others’ feelings/emotions”) to rate burnout feelings in the preceding two weeks. Item response options range from 0 (“not at all”) to 4 (“extremely”). The OBS was scored by summing up then averaging constituent item scores (minimum score = 0; maximum score = 4). OBS scores ≥ 1.33 indicated “high” burnout.

*Self-valuation* was assessed using the four-item Self-Valuation Scale (SVS) (Trockel *et al.*, 2019). It combines two items assessing deferment of self-care to prioritize work demands (e.g. “I put off taking care of my own health due to time pressure”), with two items assessing harsh responses to personal imperfections or errors (e.g. “When I made a mistake, I felt more self-condemnation than self-encouragement to learn from the experience”). Item response options are on a five-point Likert spectrum ranging from 0 (“Never”) to 4 (“Always”). The SVS is scored by summating individual items, with higher scale scores indicating greater self-valuation and vice versa. SVS scores ≥ 9 indicated “moderate-to-high” self-valuation, and SVS scores < 9 “low” self-valuation.

*Perceived autonomy support* was assessed using the six-item Physician Perceptions of Autonomy Support (PPAS-6) scale (Waddimba *et al.*, 2020). For this study, the organization was the referent on each item (e.g. “My *healthcare organization* has confidence in my ability to offer high quality care”). Item response options are on a five-point Likert spectrum ranging from 1 (“None of the time”) to 5 (“All of the time”). The PPAS-6 is scored by summating items (after reverse coding a negatively worded “interference” item) such that higher scale scores (minimum = 6; maximum = 30) indicate greater autonomy support. PPAS-6 scores ≥ 22 (i.e. 22–30) indicated perceptions of “high” support toward clinical autonomy; scores between 17 and 21 “moderate” support; and scores ≤ 17 (i.e. between 6 and 16) “low” support.

#### Contextual variables.

The multidimensional questionnaire also collected data on participants’ demographics (e.g. age, gender, race/ethnicity), service location (city), department/unit, clinical experience (years in practice) and annual patient caseload.

#### Statistical analysis strategy.

First, we screened the distribution of study variables for univariate and multivariate normality. Next, we measured internal consistency reliability of each scale/subscale using Cronbach’s coefficient alpha (Cronbach, 1951), plus ordinal coefficients alpha and theta (Zumbo *et al.*, 2007; Gadermann *et al.*, 2012). Then, we derived a polychoric correlation matrix (Holgado–Tello *et al.*, 2010) of sub/scale scores to quantify unadjusted bivariate associations between latent variables. Correlation coefficients ≥ 0.30 indicated a moderate association, and those ≥ 0.50 a strong one (Cohen, 1988).

Multivariate analyses were conducted via the two-step approach to structural equation modeling (SEM):

1. confirmatory factor analysis (CFA) of a hypothesized measurement model; and
2. extraction of the structural model with the best fit to sample data best (Anderson and Gerbing, 1988).

We used CFA/SEM as a more robust approach, rather than regression methods, to test our hypotheses as it permits specification of causal relationships between observed/indicator variables and latent constructs while accounting for item-level measurement error (Brown, 2015). It also accommodates simultaneous testing of multiple mediator variables (Iacobucci *et al.*, 2007). To minimize missing data as a validity threat, 50 replications of the sample were generated via multiple imputations, analyses were run on each replication (Enders, 2023), then results pooled across all replicated data sets (Enders and Mansolf, 2018). A separate model was fit for each of two exogenous latent variables: professional fulfillment and overall burnout. The comparative fit index (CFI) (Hu and Bentler, 1999), Tucker–Lewis index (TLI) (Hu and Bentler, 1999) and standardized root mean squared residual (SRMR) (Hu and Bentler, 1999; Maydeu-Olivares, 2017; Shi *et al.*, 2020) were used to evaluate each model’s overall fit to the data. Threshold values of CFI ≥ 0.95, TLI ≥ 0.95 and SRMR ≤ 0.08 indicated “good” data fit. Statistical analyses were conducted using SAS version 9.4 (SAS Inc, Cary, NC) and Mplus version 8.11 (Muthén and Muthén, Los Angeles, CA). CFA/SEM models used the diagonally weighted least-squares (DWLS) estimator in SAS and weighted least squares, mean- and variance-adjusted (WLSMV) estimator in Mplus.

## Results

### Sample characteristics

We solicited 815 eligible participants, of whom 122 responded at least partially (response rate ≈ 14.97%), and 99 (12.15%) responded to every question. Respondents predominantly were male (75.76%), aged 41 to < 65 years (54.54%), white (44.44%) or Asian (21.21%) and practiced at one of the hospitals and/or affiliated clinics (78.79%). A plurality (41.41%) belonged to a regional physician provider network. The majority (52.52%) had practiced for ≥ 15 years. Median (Q1, Q3) annual caseload was 250 (50, 600) patient-care encounters per year. About one-third (35.35%) were (non-invasive/interventional) cardiologists, whereas cardiovascular surgeons (11.11%) and anesthesiologists (11.11%) were the next two most self-reported specialties. Table 1 outlines the characteristics of the respondents’ sample.

**Table 1.** Social demographics and clinical work characteristics of the study sample

Variable/characteristic	Study sample (*n* = 99)
*Gender, *n* (%)*	
• Male	75 (75.76)
• Female	12 (12.12)
• Prefer not to answer	12 (12.12)
*Age group, *n* (%)*	
• 31–40 years	16 (16.16)
• 41–50 years	24 (24.24)
• 51–64 years	30 (30.30)
• ≥ 65 years	8 (8.08)
• Prefer not to answer	21 (21.21)
*Race/ethnicity^a^, *n* (%)*	
• White/Non-Hispanic	44 (44.44)
• Prefer not to answer	23 (23.23)
• Asian	21 (21.21)
• Middle Eastern or North African	3 (3.03)
• Asian Indian/Indian American	3 (3.03)
• Hispanic or Latinx/Latino/Latina	2 (2.02)
• Black/African American	2 (2.02)
• American Indian/Alaskan Native	1 (1.01)
• Other	1 (1.01)
*Specialty/department, *n* (%)*	
• Non-invasive cardiology	19 (19.19)
• Interventional cardiology	16 (16.16)
• Cardiovascular surgery	11 (11.11)
• Anesthesia	11 (11.11)
• Other	14 (14.14)
• Electrophysiologist (EP)	7 (7.07)
• Hospitalist	7 (7.07)
• Radiology	7 (7.07)
• Emergency medicine	7 (7.07)
*Clinical practice experience, *n* (%)*	
• 1–5 years	11 (11.11)
• 6–10 years	19 (19.19)
• 11–15 years	12 (12.12)
• 16–20 years	15 (15.15)
• > 20 years	37 (37.37)
• Prefer not to answer	5 (5.05)
*Patient case volume, median (Q1, Q3)*	
• Count of patient encounters per year	250 (50, 600)

**Source(s):** Authors’ own work

### Distribution and reliability of study measures

The PPAS-6 measure (skewness = –0.891; kurtosis = 1.273), and not the other scales, had a univariate kurtosis value exceeding |1.0|. Values for Cronbach’s coefficient alpha, plus ordinal coefficients alpha and theta were ≥ 0.700 for all the sub/scales, indicating that they were very reliable measures of their latent constructs. Table 2 details the descriptive statistics for the study measures. Of all respondents, 63.64% reported high professional fulfillment, 24.24% high overall burnout, 70.71% high autonomy support from the organization and 55.56% high self-valuation.

**Table 2.** Descriptive statistics, variability, dispersion and reliability of scores on the study measures

Construct assessed	Measure (subscale or scale) utilized	No. of items	Mean score (± SD)	Skewness	Kurtosis	Ordinal coefficient alpha (ρ)	Ordinal coefficient theta (θ)	Cronbach’s coefficient alpha^∫^ (α)
Well-being-centered support	Adapted version of the Mayo	9	34.33 (± 9.09)	–0.722	–0.030	0.973	0.973	0.957
From a leader	Leadership Impact Index							
Organizational support for	Physician Perceptions of	6	22.55 (± 3.38)	–0.891	1.273	0.934	0.0.935	0.898
Physicians’ clinical autonomy	Autonomy Support (PPAS)							
Physicians’ self-valuation and self-care	Self-Valuation Scale (SVS)	4	9.32 (± 3.71)	0.089	–0.488	0.857	0.857	0.821
Physicians’ overall	Overall Burnout Scale (OBS)	10	0.83 (± 0.72)	0.730	–0.362	0.956	0.957	0.933
Burnout								
Physicians’ professional	Professional Fulfillment	6	2.93 (± 0.82)	–0.828	0.366	0.948	0.948	0.922
Fulfillment	Scale							
	(PFS)							

**Note(s):** SD = standard deviation; ∫ = standardized Cronbach’s coefficient alpha

**Source(s):** Authors’ own work

### Univariate associations between study measures

Table 3 depicts the polychoric correlation matrix between the measures. Burnout had high, negative correlations with fulfillment (–0.689), self-valuation (–0.687), plus autonomy support (–0.536) and a moderate, negative correlation with well-being-centered leadership (–0.398). Fulfillment had strong, positive correlations with autonomy support (0.574) and leader ratings (0.510) and a moderate one with self-valuation (0.446). Self-valuation had moderate, positive correlations with autonomy support (0.443) and leader ratings (0.349); autonomy support a high, positive correlation (0.610) with leader ratings.

**Table 3.** Polychoric correlation matrix of the latent variable measures

#	Latent construct or trait					
		1	2	3	4	5
		Leadership support	Autonomy support	Self-valuation and self-care	Overall burnout	Professional fulfillment
1	Well-being-centered leadership support	1.000				
2	Physician Perceptions of Autonomy Support	0.6103^ǂ^	1.000			
3	Self-Valuation and Self-Care	0.3492^*^	0.4432^+^	1.000		
4	Overall burnout	−0.3981^*^	−0.5364^ǂ^	−0.6870^ǂ^	1.000	
5	Professional fulfillment	0.5099^ǂ^	0.5736^ǂ^	0.4455^+^	−0.6885^ǂ^	1.000

**Note(s):**

*p < 0.05;

^+^ *p* < 0.01;

ǂ*p* < 0.001

**Source(s):** Authors’ own work

### Multivariate analysis findings

The hypothesized mediated model of professional fulfillment showed good overall fit to the data (CFI = 0.977, TLI = 0.974, SRMR = 0.061), as did that of overall burnout (CFI = 0.971, TLI = 0.968, SRMR = 0.067). The fulfillment model accounted for large proportions of the variance in fulfillment (*R*^2^ = 0.464, or 46.4%; *p* < 0.001) and autonomy support (*R*^2^ = 0.588, or 58.8%; *p* < 0.001), but a smaller proportion of variance in self-valuation (*R*^2^ = 0.126, or 12.6%; *p* = 0.046). Likewise, the burnout model accounted for large proportions of variance in burnout (*R*^2^ = 0.609, or 60.9%; *p* < 0.001) and autonomy support (*R*^2^ = 0.588, or 58.8%; *p* < 0.001), but a smaller proportion of variance in self-valuation (*R*^2^ = 0.126, or 12.6%; *p* = 0.047).

**Figure 1.**
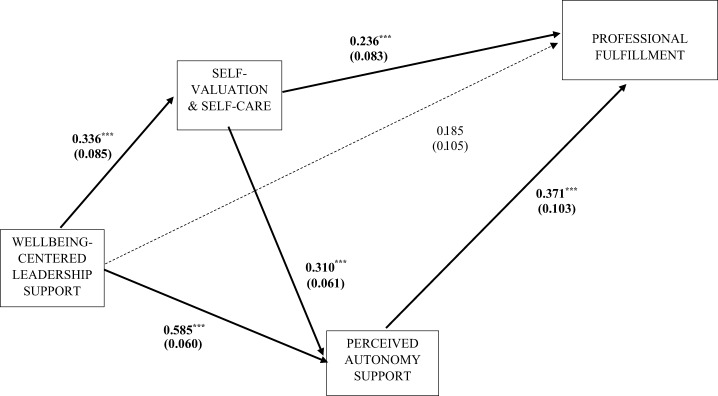
Structural model of the association of well-being-centered leadership with professional fulfillment **Sources:** Authors’ own work

The total effects (direct plus indirect effects) in the mediated model of fulfillment were statistically significant (overall standardized coefficient (*β_standardized_*) = 0.545; *p* < 0.001). The combined indirect effects specified in all the mediated pathways collectively were significant (*β* = 0.351; *p* < 0.001), but the direct effects in the model did not reach overall significance (*β* = 0.194; *p* = 0.074). As shown in Table 4, all three endogenous variables in the model (i.e. leader ratings, self-valuation and autonomous support) had significant indirect effects on fulfillment, totaling three significant mediated pathways from well-being-centered leadership to fulfillment. As shown in Figure 1, leader ratings had significant direct effects on self-valuation (*β* = 0.336; *p* < 0.001) and autonomy support (*β* = 0.585; *p* < 0.001). However, direct effects of leader ratings on professional fulfillment were not significant (*β* = 0.185; *p* > 0.05). Self-valuation had significant direct effects on autonomy support (*β* = 0.236; *p* < 0.001) and fulfillment (*β* = 0.310; *p* < 0.001), whereas autonomy support had significant direct effects on fulfillment (*β* = 0.371; *p* < 0.001). Findings suggest that the proportion of the influence of well-being-centered leadership on professional fulfillment that is mediated through self-valuation and autonomy support is more substantial than any direct effects. The direct association of leadership support with fulfillment is not significant once effects mediated via self-valuation and autonomy support are accounted for.

**Table 4.** Standardized total, direct and indirect effects of well-being-centered leadership on professional fulfillment with self-valuation and perceived autonomy support as Mediators^Σ^

Structural model pathway	Standardized coefficient (*β*)	95% confidence interval	z-statistic	Significance (*p*-value)
*Total effects*				
Leader support → Fulfillment	*0.545*	*0.434–0.657*	*8.055*	*<0.001*
*Direct effects*				
Leader support → Fulfillment	0.194	0.015–0.373	1.787	0.074
*Total indirect effects*				
Leader support → Fulfillment	*0.351*	*0.226–0.477*	*4.607*	*<0.001*
*Specific indirect effects*				
Leader support → Self-valuation → Fulfillment	*0.083*	*0.027–0.139*	*2.451*	*0.014*
Leader support → Autonomy support → Fulfillment	*0.228*	*0.111–0.345*	*3.205*	*0.001*
Leader support → Self-valuation → Autonomy support → Fulfillment	*0.041*	*0.018–0.063*	*2.941*	*0.003*

**Note(s):** Σ = results are averaged across 50 multiply imputed replications of the study sample, italicized results are statistically significant

**Source(s):** Authors’ own work

**Figure 2.**
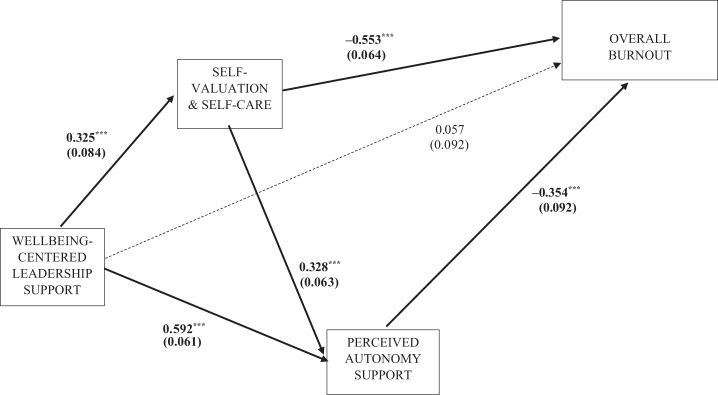
Structural model of the association of well-being-centered leadership with overall burnout **Source:** Authors’ own work

The total effects (direct plus indirect effects) in the mediated model of burnout were also significant (overall *β_standardized_* = −0.401; *p* < 0.001). Once again, the combination of indirect effects in all mediated pathways collectively were significant (*β* = –0.463; *p* < 0.001), but the direct effects specified in the model did not reach overall significance (*β* = 0.062; *p* = 0.538). As shown in Table 5, all three endogenous variables (i.e. leader ratings, self-valuation and autonomous support) had significant indirect effects on burnout. As shown in Figure 2, leader ratings had significant direct effects on self-valuation (*β* = 0.325; *p* < 0.001) and autonomy support (*β* = 0.592; *p* < 0.001), whereas direct effects of leader ratings on burnout were insignificant (*β* = 0.057; *p* > 0.05). Self-valuation had significant direct effects on autonomy support (*β* = 0.328; *p* < 0.001) and on burnout (β = –0.553; *p* < 0.001), while autonomy support had significant direct effects on burnout (*β* = –0.354; *p* < 0.001). The direct association of leadership support with burnout is not significant once effects mediated via self-valuation and autonomy support are accounted for.

**Table 5.** Standardized total, direct and indirect effects of well-being-centered leadership on overall burnout with self-valuation and perceived autonomy support as Mediators^Σ^

Structural model pathway	Standardized coefficient (*β*)	95% confidence interval	z-statistic	Significance (*p*-value)
*Total effects*				
Leader support → Burnout	*−0.401*	*−0.540 to –0.463*	*−4.772*	*<0.001*
*Direct effects*				
Leader support → Burnout	0.062	−0.103–0.226	0.100	0.538
*Total indirect effects*				
Leader support → Burnout	*−0.463*	*−0.602 to –0.324*	*−5.477*	*<0.001*
*Specific indirect effects*				
Leader support → Self-valuation → Burnout	*−0.195*	*−0.283 to –0.106*	*−3.630*	*<0.001*
Leader support → Autonomy support → Burnout	*−0.227*	*−0.336 to –0.119*	*−3.438*	*0.001*
Leader support → Self-valuation → Autonomy support → Burnout	*−0.041*	*−0.062 to –0.019*	*−3.090*	*0.002*

**Note(s): **Σ = results are averaged across 50 multiply imputed replications of the study sample, italicized results are statistically significant

**Source(s):** Authors’ own work

## Discussion

The present study investigated the extent to which intrinsic self-valuation and extrinsic autonomy support mediated the association of well-being-centered leadership support with professional fulfillment and overall burnout among physicians. In a cross-sectional observational study of physicians practicing within a three-hospital health-care system, we adduced evidence that self-valuation and autonomy support partially mediate the effects of leader support ratings on the two study outcomes. Direct associations of leadership support with fulfillment and burnout were not significant after accounting for indirect effects mediated via self-valuation and autonomy support in the multivariate pathways analyzed. Findings suggest that well-being-centered leadership improves burnout and fulfilment via mechanisms such as fostering autonomy support, challenging a perfectionist mindset and encouraging self-care, self-compassion, a healthy acceptance of imperfections and willingness to learn from errors.

Challenges to prioritization of self-valuation/self-care by physicians are deeply embedded in their workplace culture (Trockel *et al.*, 2021; Shanafelt *et al.*, 2019a), and it appears support from a leader or other person or group of influence may give physicians leverage to act as organizational culture change agents (Feussner *et al.*, 2016; Gonzalo *et al.*, 2020). Leaders wield oversight over decisions about scheduling, clinical workflow, quality improvement initiatives and can be influential in challenging intra-personal attitudes of prioritizing patient care above self-care. Indeed, prioritizing personal well-being and a positive growth mindset has been shown to account for up to 27% of the variability in burnout (Trockel *et al.*, 2019). There is early evidence of the effectiveness of coaching programs in improving self-valuation and well-being among physicians (Makowski *et al.*, 2022; Smith *et al.*, 2024). One randomized controlled trial of peer coaching reported an improvement in self-valuation that fell just short of the significance threshold (Kiser *et al.*, 2024). Future studies should further explore how health-care leaders can spearhead new, theory-informed approaches to enhancing a culture of self-valuation as an intervention strategy aimed at improving fulfillment and reducing burnout among physicians.

The findings also supported our hypothesis that perceived autonomy support from the organization significantly mediates the influence of leadership behavior on both fulfillment and burnout among physicians. Autonomy support is a key ingredient in the professional well-being of practicing physicians (Moreau and Mageau, 2012; Lases *et al.*, 2018), as well as in healthy mentoring of trainee physicians (Sawatsky *et al.*, 2022). Well-being-centered leadership aims to empower rather than overpower and to support rather than suppress physicians’ clinical autonomy (Montgomery, 2016; Slemp *et al.*, 2018). Support toward autonomy motivates adherence to evidence-based practice (Waddimba *et al.*, 2019). Physicians receiving greater autonomy support are more likely to perceive their organization as central to their professional identity (Salvatore *et al.*, 2018).

Well-being-centered leadership training incorporates skills such as caring for physicians (which includes fostering self-valuation), cultivating relationships and a shared sense of purpose within teams and inspiring culture change plus operational improvements (Hopkins *et al.*, 2018; Shanafelt *et al.*, 2021b; American Medical Association, 2024). Trainings involving multiple components can be expensive and time-consuming, and the source of any benefits often remains unclear (Straus *et al.*, 2013). Our findings identify specific factors that leadership training can target. Leaders can be effective role models by personally demonstrating well-being and self-care behaviors (Shanafelt *et al.*, 2020). Thus, training that improves their own self-valuation and autonomy support could make leaders better change agents. Training should also equip leaders with the skills of mentoring teammates to support one another’s self-care and autonomy. This study adds to our understanding of how well-being-centered leadership benefits physicians. Future studies should investigate additional mediators of the effect(s) of leadership behavior on physician well-being, especially in systems with matrixed structures where a single, direct-report supervisor over each physician is not the norm. Other potential mediators might include such factors as teamwork and/or values alignment.

The present study had notable limitations. First, the cross-sectional design precludes considerations of causation or the temporal sequence of the factors investigated. Second, the study was confined to physicians in one health system, limiting the generalizability of results. Third, the modest sample size limited the statistical power. Fourth, due to the strict anonymity of survey, we had no data on non-responders and were unable to assess response bias. Fifth, the study focused on just two of numerous potential mediators of the effect of leadership behavior on physician well-being. Strengths of the study include high reliability and validity of the ordinal scales, plus good overall fit of hypothesized models to the study data. Findings must be interpreted with caution as preliminary evidence. These should be replicated in prospective studies on larger samples of physicians affiliated with multiple health-care delivery systems, belonging to diverse clinical sub/specialties and serving a variety of patients in disparate geolocations.

## Conclusion

Well-being-centered leadership behaviors have been found, in numerous studies, to be associated with burnout and professional fulfillment among physicians. Despite this evidence, little is known about exact mechanisms of action through which such leadership support impacts occupational well-being. Our findings suggest that empowering physicians to practice according to their professional volition plus encouraging self-care and a positive growth mindset are, in part, the mechanisms by which leadership behavior impacts well-being. Health-care leaders who cultivate environments characterized by clinical autonomy in which self-care is encouraged and enabled are effective at improving physician well-being. Well-being-centered leadership behaviors appear to result in environments that provide more functional support toward practice autonomy as well as emotional support towards self-valuation.
